# Breastfeeding self-efficacy in adult women and its relationship with exclusive maternal breastfeeding

**DOI:** 10.1590/1518-8345.3652.3364

**Published:** 2020-09-30

**Authors:** Juliana Cristina dos Santos Monteiro, Carolina Maria de Sá Guimarães, Luciana Camargo de Oliveira Melo, Marina Cortez Pereira Bonelli

**Affiliations:** 1 Universidade de São Paulo, Escola de Enfermagem de Ribeirão Preto, PAHO/WHO Collaborating Centre for Nursing Research Development, Ribeirão Preto, SP, Brazil.; 2 Scholarship holder at the Coordenação de Aperfeiçoamento de Pessoal de Nível Superior (CAPES), Brazil.

**Keywords:** Breastfeeding, Self Efficacy, Obstetric Nursing, Maternal and Child Health, Women’s Health, Child Health, Aleitamento Materno, Autoeficácia, Enfermagem Obstétrica, Saúde Materno-Infantil, Saúde da Mulher, Saúde da Criança, Lactancia Materna, Autoeficacia, Enfermería Obstétrica, Salud Materno-Infantil, Salud de la Mujer, Salud del Niño

## Abstract

**Objective:**

to analyze the relationship between maternal self-efficacy to breastfeed and sociodemographic, obstetric, and neonatal variables; between the duration of exclusive breastfeeding and sociodemographic variables; and between the breastfeeding self-efficacy and the duration of exclusive breastfeeding at the intervals of 30, 60, and 180 days postpartum.

**Method:**

a longitudinal and prospective study conducted with 224 women. A sociodemographic questionnaire, the Breastfeeding Self-Efficacy Scale – Short Form, and a questionnaire on breastfeeding and child feeding were used for collecting the data. Fisher’s exact test and Pearson’s correlation coefficient test were used for analysis.

**Results:**

there was no association between breastfeeding self-efficacy and the duration of exclusive breastfeeding identified at 30, 60, and 180 days. Self-efficacy was associated with the type of delivery and complications in the postpartum period. There was also an association between religion and exclusive breastfeeding 30 and 60 days postpartum, and assistance with baby care and exclusive breastfeeding at 60 days.

**Conclusion:**

It was identified that the type of delivery, complications in the postpartum period, religion, and assistance with baby care corroborate to increase maternal confidence in the ability to breastfeed.

## Abstract (pt)

**Objetivo:**

analisar a relação entre a autoeficácia materna para amamentar e as variáveis sociodemográficas, obstétricas e neonatais; a duração do aleitamento materno exclusivo e as variáveis sociodemográficas, e a autoeficácia para amamentar e a duração do aleitamento materno exclusivo nos intervalos de 30, 60 e 180 dias pós-parto.

**Método:**

estudo longitudinal e prospectivo realizado com 224 mulheres. Para coleta de dados, utilizou-se um questionário sociodemográfico, a *Breastfeeding Self-Efficacy Scale* – *Short Form* e um questionário sobre amamentação e alimentação da criança. Para análise utilizou-se o Teste Exato de Fisher e o Coeficiente de Correlação de Pearson.

**Resultados:**

identificou-se que não houve associação entre a autoeficácia para amamentar e a duração do aleitamento materno exclusivo em 30, 60 e 180 dias. A autoeficácia apresentou associação com o tipo de parto e intercorrência no pós-parto. Também se encontrou associação entre a religião e o aleitamento materno exclusivo com 30 e 60 dias pós-parto, e auxílio aos cuidados com o bebê e o aleitamento materno exclusivo em 60 dias.

**Conclusão:**

identificou-se que o tipo de parto, intercorrência no pós-parto, religião e auxílio aos cuidados com o bebê corroboram para incrementar a confiança materna na capacidade para amamentação.

## Abstract (es)

**Objetivo:**

analizar la relación entre la autoeficacia materna para amamantar y las variables sociodemográficas, obstétricas y neonatales; la duración del amamantamiento materno exclusivo y las variables sociodemográficas, y la autoeficacia para amamantar y la duración de la lactancia materna exclusiva a intervalos de 30, 60 y 180 días posparto.

**Método:**

estudio longitudinal y prospectivo realizado con 224 mujeres. Para la recolección de datos, se utilizó un cuestionario sociodemográfico, la Breastfeeding Self-Efficacy Scale – Short Form y un cuestionario sobre amamantamiento y alimentación infantil. La Prueba Exacta de Fisher y el coeficiente de correlación de Pearson se utilizaron para el análisis.

**Resultados:**

se identificó que no hubo asociación entre la autoeficacia para amamantar y la duración de la lactancia materna exclusiva en 30, 60 y 180 días. La autoeficacia se asoció con el tipo de parto y las intercurrencias en el período posparto. También hubo una asociación entre la religión y la lactancia materna exclusiva con 30 y 60 días posparto, y entre la asistencia en los cuidados del bebé y la lactancia materna exclusiva en 60 días

**Conclusión:**

se identificó que el tipo de parto, las intercurrencias en el período posparto, la religión y la asistencia en los cuidados del bebé corroboran para aumentar la confianza materna en la capacidad de amamantar.

## Introduction

The contributions of breastfeeding are already known worldwide, and its benefits are extended not only to the health of women and children, but also to the whole family and society^(^ 1 ^)^ and, due to these contributions, the World Health Organization (WHO) and the Brazilian Ministry of Health (MoH) recommend exclusive breastfeeding (EBF) until the child’s sixth month of life^(^ 2 ^)^. Despite all the benefits described in the scientific literature, only 37% of the children born in Brazil are exclusively breastfed until the sixth month of life, a rate below the one recommended by the WHO, which is at least 50%^(^ 2 ^-^ 4 ^)^.

The breastfeeding practice is a complex process that goes beyond biological determinism, and the woman’s decision to breastfeed or not also involves social, cultural, economic, and psychological factors^(^ 5 ^)^. A study carried out in the Federal District (Brazil) found that the causes for early weaning were related to the beliefs of weak milk and to the women’s need to return to work^(^ 6 ^)^. Another study carried out in Valencia (Spain) observed that there was a relationship with breastfeeding time and maternal age, parity, low milk production, and low weight gain of the baby^(^ 7 ^)^.

The support received by women during this process is also a factor that strongly influences the practice of breastfeeding, whether such support is received by family members, friends, or health professionals^(^ 8 ^)^. In addition, today women also seek support through social networks, websites, and Internet pages that provide information on breastfeeding practices, being a new type of health intervention^(^ 9 ^)^.

Some research studies indicate that early interruption of breastfeeding can be related to the low confidence of women in their ability to breastfeed their babies, which is an important variable that influences the initiation and maintenance of breastfeeding^(^ 10 ^-^ 11 ^)^. Maternal confidence in the ability to breastfeed is also known as breastfeeding self-efficacy, and can be modified through individual interventions with women^(^ 10 ^)^.

The construct of breastfeeding self-efficacy construct consists of four sources of information, which directly influence women’s decision to start and maintain this practice: by the woman’s personal experience; by living with other breastfeeding women (vicarious experience); by the information received both from their social support network and from the health professional who accompanies them; and by their physical and emotional condition^(^ 12 ^)^.

Those who trust their ability are known to usually breastfeed longer than those who do not have such a perception^(^ 10 ^)^. For the health professionals, who are essential in encouraging and supporting breastfeeding, the identification of mothers who are at risk for early weaning (based on modifiable variables), such as low breastfeeding self-efficacy, can facilitate the development and evaluation of interventions that favor breastfeeding^(^ 13 ^-^ 14 ^)^.

Brazil is a country with great cultural and economic diversity, so understanding the behavior of women in different scenarios can provide subsidies for the improvement of public policies to promote and protect breastfeeding. Thus, the objectives of this study were to analyze the relationship between maternal breastfeeding self-efficacy and sociodemographic, obstetric, and neonatal variables; the duration of exclusive breastfeeding and sociodemographic variables, and the breastfeeding self-efficacy and the duration of exclusive breastfeeding at the intervals of 30, 60, and 180 days postpartum.

## Method

A longitudinal and prospective study conducted on 224 randomly selected women, following these inclusion criteria: being 18 years old or older, with at least 24 hours postpartum, in sound physical condition to breastfeed, accompanied by their children in joint accommodation, who had children of full term gestational age, and who had a landline or cell phone. The exclusion criteria were the following: women with hearing and/or visual impairment, disoriented in terms of time, space or person, with newborns who were receiving special care and/or who had an anomaly or malformation.

The study was carried out in Ribeirão Preto, from January 2014 to June 2015, in two stages. The first stage was carried out in the joint accommodation, where women who met the inclusion criteria and agreed to participate in the study after being instructed on it signed the Free and Informed Consent Form. Two data collection instruments were applied, with a mean duration of 15 minutes for completion: the first instrument included sociodemographic (age, self-reported skin color, schooling, religion, marital status, income, and assistance with baby care) and obstetric data (number of children, current pregnancy planning, number of prenatal consultations, type of delivery, complications in the postpartum period, gender and weight of the newborn, breastfeeding in the first hour of life of the newborn, and type of breastfeeding at the time of data collection). The second instrument applied was the Breastfeeding Self-Efficacy Scale – Short Form (BSES-SF), a Canadian scale validated in Brazil^(^ 15 ^)^, used to assess maternal confidence in the ability to breastfeed. BSES-SF is a Likert-type scale containing 14 questions divided into: Technical Domain, and Domain of Intrapersonal Thinking; each question has five possible answers ranging from 1 to 5 points, namely: 1-totally disagree, 2-disagree, 3-sometimes agree, 4-agree, 5-totally agree, and the sum of the scores varies from 14 to 70 points. Breastfeeding Self-efficacy is identified from the sum of each question: low self-efficacy (14 to 32 points), medium self-efficacy (33 to 51 points), high self-efficacy (52 to 70 points)^(^ 15 ^)^.

The second moment of the study was characterized by telephone searches carried out at 30, 60, and 180 days after the delivery, using a third data collection instrument that addressed questions regarding the type of food offered to the child (breastfeeding and/or complementary feeding), exclusive breastfeeding time in days, and what the complications were that occurred during the breastfeeding period. The telephone contact lasted an average of ten minutes and with each contact it was agreed with the woman what the best period was to make the next contact, respecting the family’s routines. Up to three telephone contact attempts were made at each moment of this stage, at different days and times and, if the participant was not found in any of the three attempts, she was excluded from the research.

The collected data were compiled and stored through double typing in a spreadsheet structured in Microsoft Excel, enabling data validation and error elimination, thus ensuring typing reliability. The data were analyzed descriptively using the Statistical Analysis System SAS^®^ 9.0 statistical program. To verify the association between the qualitative variables, the data were submitted to Fisher’s Exact Test, and the quantitative variables were categorized and also submitted to this Test. To check the relationship between maternal self-efficacy and the final breastfeeding time, the Pearson’s Correlation Coefficient was used. For all the statistical analyses, significance levels of 5% (α=0.05) were considered.

The research project was approved by the Research Ethics Committee of the Ribeirão Preto Nursing School at the University of São Paulo, with the following protocol number: 21346013.8.0000.5393.

## Results

Among the 224 (100%) investigated women, age ranged between 18 and 44 years old, with a mean of 24.65 (Standard Deviation - SD=5.79) and a median of 24 years old. With regard to the perception of their own skin color, 44.2% of the women said they were brown-skinned, 47.32% declared they had completed high school, 47.3% declared they were in love, and 79.9% reported having some religion. The majority (59.4%) reported not doing any paid work outside the home and 46.0% reported having their own home. The mean monthly family income declared was R$ 1,943.44 (one thousand, nine hundred and forty-three reais and forty-four cents), 86.6% stated that they would have help from someone to take care of the baby and, from those (n=194), 46.4% reported that help would be obtained from their own mother, 30.9% reported getting help from a partner, 11.3% referred to getting help from their mother-in-law, and 11.3% would get help from other family members and friends.

Regarding the obstetric characteristics, 47.3% of the participants were primigravidas, 50.4% were primiparous, 55.8% of the women did not plan pregnancy, 81.8% started prenatal care in the first trimester of pregnancy, 88, 7% had six or more prenatal consultations, and 77.7% had vaginal delivery (four forceps – 1.8%). Most of the women reported not having any complications during pregnancy (63.8%), labor and delivery (92.9%) or postpartum (94.6%), and 51.8% of the newborns born were male. Most of the babies (97.3%) had a weight equal to or greater than 2,500 g and 59.4% were breastfed in the first hour of life, 91.0% of the children were in EBF at the time of data collection in the maternity ward.

Regarding the assessment of breastfeeding self-efficacy, most of the women [n=188 (83.9%)] had a high level of self-efficacy, 15.6% (35) had a medium level, and 0.4% (one participant ) had a low level of self-efficacy.

The analysis of the association between self-efficacy and the sociodemographic variables (age, self-reported skin color, schooling, religion, marital status, income, and assistance with baby care) did not show a statistically significant result (Table 1).

**Table 1:** Breastfeeding Self-efficacy associated with the participants' sociodemographic characteristics (n=224). Ribeirão Preto, SP, Brazil, from 2014 to 2015

Self-efficacy					
	Low n (%)	Moderate n (%)	High n (%)	Total n (%)	*p*-value*
Age group					
Up to 24 years old	0 (0.0)	21 (9.4)	94 (42.0)	115 (51.3)	0.4958
25 to 34 years old	1 (0.4)	12 (5.4)	84 (37.5)	97 (43.3)	
35 years old or more	0 (0.0)	2 (0.9)	10 (4.5)	12 (5.4)	
Total	1 (0.4)	35 (15.6)	188(83.9)	224 (100.0)	
Self-reported skin color					
White	1 (0.4)	9 (4.0)	75 (33.5)	85 (37.9)	0.1727
Black/Brown	0 (0.0)	24 (10.7)	107(47.8)	131 (58.5)	
Asian	0 (0.0)	2 (0.9)	6 (2.7)	8 (3.6)	
Total	1 (0.4)	35 (15.6)	188(83.9)	224 (100.0)	
Schooling					
Incomplete Primary	0 (0.0)	4 (1.8)	19 (8.5)	23 (10.3)	0.6065
Complete Primary	0 (0.0)	12 (5.4)	77 (34.4)	89 (39.7)	
Complete Secondary	1 (0.4)	17 (7.6)	88 (39.3)	106 (47.3)	
Complete Higher Education	0 (0.0)	2 (0.9)	4 (1.8)	6 (2.7)	
Total	1 (0.4)	35 (15.6)	188(83.9)	224 (100.0)	
Religion					
Has a religion	1 (0.4)	28 (12.5)	150(67.0)	179 (79.9)	1.0000
Atheist/Agnostic	0 (0.0)	7 (3.1)	38 (17.0)	45 (20.1)	
Total	1 (0.4)	35 (15.6)	188(83.9)	224 (100.0)	
Marital status					
No partner	0 (0.0)	8 (3.6)	42 (18.7)	50 (22.3)	1.0000
Has a partner	1 (0.4)	27 (12.0)	146 (65.2)	174 (77.7)	
Total	1 (0.4)	35 (15.6)	188 (83.9)	224 (100.0)	
Income in MWs^†^					
Up to 1.9	1 (0.5)	14 (7.0)	87 (43.5)	102 (51.0)	0.9749
From 2 to 3.9	0 (0.0)	13 (6.5)	70 (35.0)	83 (41.5)	
4 or more	0 (0.0)	2 (1.0)	13 (6.5)	15 (7.5)	
Total	1 (0.5)	29 (14.5)	170 (85.0)	200 (100.0)	
Baby Care Help					
Yes	1 (0.4)	31 (13.8)	162 (72.3)	194 (86.6)	1.0000
No	0 (0.0)	4 (1.8)	26 (11.6)	30 (13.4)	
Total	1 (0.4)	35 (15.6)	188 (83.9)	224 (100.0)	

* Fisher's Exact Test;

† MWs = Minimum wages. Brazilian minimum wage in 2016: R$ 880.00

As for the obstetric characteristics, the “type of delivery” and “complications in the postpartum” variables showed a statistically significant association with self-efficacy. Women who had a vaginal delivery had a higher level of self-efficacy than those who underwent cesarean section (p=0.0376). The women who had no complications in the postpartum period showed greater self-efficacy than those who had complications (p=0.0410). These results are described in Table 2.

**Table 2:** Breastfeeding Self-efficacy, associated with the participants' obstetric characteristics (n=224). Ribeirão Preto, SP, Brazil, from 2014 to 2015

Self-efficacy					
	Low n (%)	Moderate n (%)	High n (%)	Total n (%)	*p*-value*
Number of children					
One	0 (0.0)	21 (9.4)	95(42.4)	116 (51.8)	0.3155
Two or more	1 (0.4)	14 (6.2)	93 (41.5)	108 (48.2)	
Total	1 (0.4)	35 (15.6)	188 (83.9)	224(100.0)	
Planned pregnancy					
Yes	0 (0.0)	10 (4.5)	89 (39.7)	99 (44.2)	0.0519
No	1 (0.4)	25 (11.2)	99 (44.2)	125 (55.8)	
Total	1 (0.4)	35 (15.6)	188 (83.9)	224(100.0)	
Number of prenatal consultations					
Up to 5	0 (0.0)	4 (1.9)	20 (9.4)	24 (11.3)	1.0000
6 or more	1 (0.4)	30 (14.1)	158 (74.2)	189 (88.7)	
Total	1 (0.4)	34 (16.0)	178 (83.6)	213(100.0)	
Type of delivery					
Vaginal	0 (0.0)	31 (13.8)	143 (63.8)	174 (77.7)	0.0376
Cesarean	1 (0.4)	4 (1.8)	45 (20.1)	50 (22.3)	
Total	1 (0.4)	35 (15.6)	188 (83.9)	224(100.0)	
Postpartum complication					
Yes	1 (0.4)	2 (0.9)	9 (4.0)	12 (5.4)	0.0410
No	0 (0.0)	33 (14.7)	179 (79.9)	212 (94.6)	
Total	1 (0.4)	35 (15.6)	188 (83.9)	224(100.0)	

* Fisher's Exact Test

Table 3 shows the results of the association between maternal self-efficacy and the variables related to the characteristics of the participants’ children and breastfeeding. No statistically significant associations were found for these analyses.

**Table 3:** Breastfeeding Self-efficacy associated with the characteristics of the participants' children (n=224). Ribeirão Preto, SP, Brazil, from 2014 to 2015

Self-efficacy					
	Low n (%)	Moderate n (%)	High n (%)	Total n (%)	*p*-value*
Gender of the child					
Female	0 (0.0)	19 (8.5)	89 (39.7)	108 (48.2)	0.5228
Male	1 (0.4)	16 (7.1)	99 (44.2)	116 (51.8)	
Total	1 (0.4)	35 (15.6)	188 (83.9)	224(100.0)	
Birth weight					
<2,500 grams	0 (0.0)	0 (0.0)	6 (2.3)	6 (2.7)	0.6041
≥2,500 grams	1 (0.4)	35 (15.6)	182 (81.2)	218 (97.3)	
Total	1 (0.4)	35 (15.6)	188 (83.9)	224(100.0)	
Breastfeeding in the first hour of life					
Yes	0 (0.0)	18 (8.0)	115(51.3)	133 (59.4)	0.2239
No	1 (0.4)	16 (7.1)	71 (31.7)	88 (39.3)	
Does not know	0 (0.0)	1 (0.4)	2 (0.9)	3 (1.3)	
Total	1 (0.4)	35 (15.6)	188 (83.9)	224(100.0)	
Type of breastfeeding					
Exclusive breastfeeding	1 (0.4)	31 (13.8)	172 (76.8)	204 (91.1)	
Breastfeeding with other fluids/milk	0 (0.0)	4 (1.8)	16 (7.1)	20 (8.9)	
Total	1 (0.4)	35 (15.6)	188 (83.9)	224 (100.0)	

* Fisher's Exact Test

Regarding the duration of the EBF, at 30 days postpartum, 64.4% (136) of the women breastfed exclusively, at 60 days, 55.6% (109) breastfed exclusively and only 6.0% (9) of the women maintained EBF up to 180 days postpartum.

The association between the sociodemographic variables and the duration of breastfeeding showed a statistically significant result between EBF 30 and 60 days postpartum and the “religion” variable (p=0.0367 and 0.0073, respectively), showing that the maintenance of exclusive breastfeeding at 30 and 60 days was higher among women who reported having some religion. There was also a statistically significant association between EBF at 60 days and the assistance in baby care variable (p=0.0324), that is, the maintenance of exclusive breastfeeding at 60 days was greater among the participants who reported having this assistance.

The investigation of associations between the breastfeeding self-efficacy variable and the duration of EBF in the periods of 30, 60 and 180 days after delivery did not present a statistically significant result by Fisher’s exact test (p=0.6328, p=0.0798, and p=1.0000, respectively).

**Figure 1:**
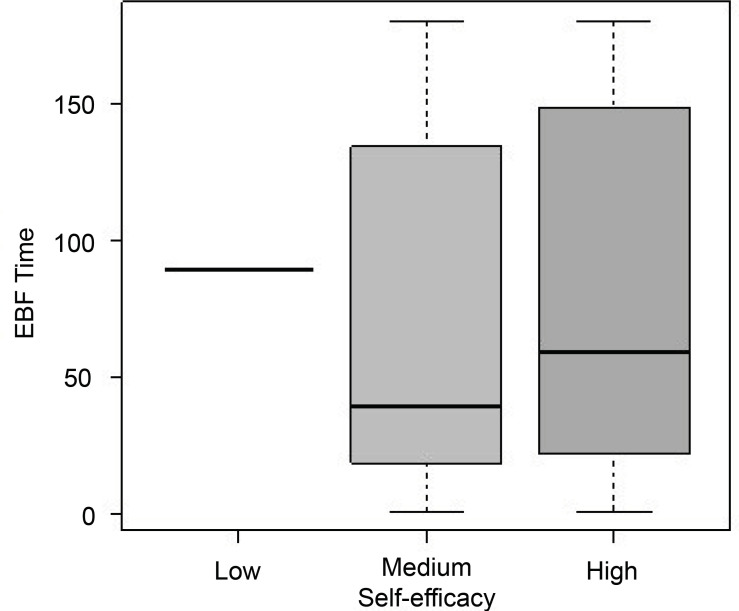
Distribution of the final EBF time in days, according to the classification of breastfeeding self-efficacy

The total EBF time, in days, is shown in Figure 1. The only participant who had low self-efficacy breastfed for 89 days. Among the participants who had a medium level of self-efficacy, the mean EBF duration was 69.8 days. Among those with a high level of self-efficacy, the mean EBF time was 82.49 days.

**Figure 2:**
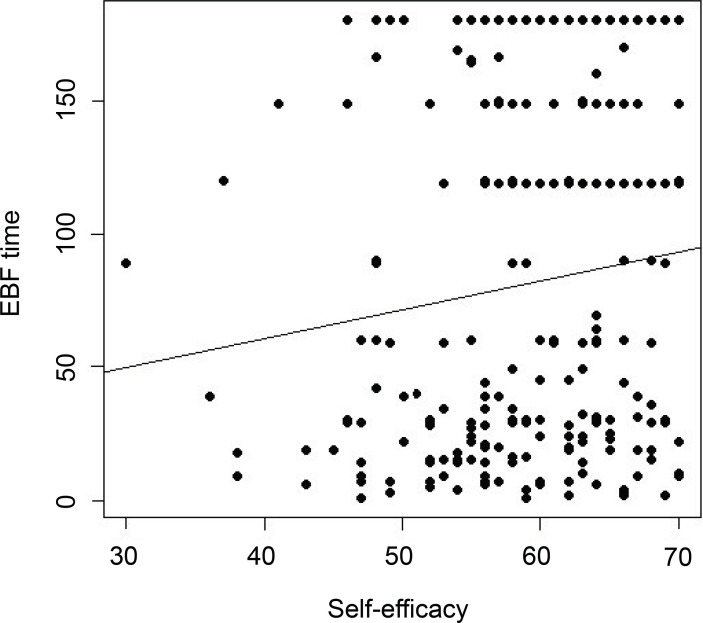
Correlation between breastfeeding self-efficacy and EBF final time in days

Pearson’s Correlation analysis between self-efficacy and final breastfeeding time was performed, with a p-value of 0.0640, and the correlation coefficient was 0.122396. The results show that there was no statistically significant relationship between these two variables. Figure 2 illustrates this result, drawing attention to a positive, but weak, relationship between the two variables.

## Discussion

The value of self-efficacy among the study participants pointed out that most of them (83.9%) had a high level of this variable, with the mean of the BSES-SF total score being 58.62 points, corroborating with some studies carried out in the national and international scopes^(^ 16 ^-^ 18 ^)^. The present study showed a significant association between breastfeeding self-efficacy and the type of delivery, confirming studies carried out in the Philippines^(^ 18 ^)^ and in the Northeastern region of Brazil^(^ 19 ^)^. It also showed a significant association between self-efficacy and the postpartum complications variable. No scientific studies were found that analyzed the relationship between self-efficacy and complications during pregnancy, labor and delivery, and postpartum. However, this analysis leads us to the fourth source of information on the self-efficacy construct (emotional and physiological state), since they are relevant data for the health professionals, when they also use verbal persuasion (third source of information on the self-efficacy construct). In this way, the professionals can have a more accurate performance in supporting women during breastfeeding, according to the demand of each one of them^(^ 8 ^)^.

The final mean EBF duration among the participants was 80.54 days and the median was 59 days and, at the three moments investigated, there was a decrease in the percentage of women who maintained EBF: 64.4% at 30 days, 55.6% at 60 days, and 6.0% at 180 days, being below the indexes in children under six months presented in the world, which is 35.7%, according to a study published in 2016^(^ 1 ^)^. The fact that the study site is accredited by the Baby-Friendly Hospital Initiative (BFHI) and has institutional actions in favor of breastfeeding did not guarantee that the women kept EBF outside the hospital setting according to the official recommendations. In this sense, it can be inferred that the high level of self-efficacy was essential for the practice of breastfeeding initiated and carried out in the maternity ward; however, when returning to their homes, it is observed that breastfeeding did not depend only on the mastery of management techniques and on the desire shown in the hospital setting. Internal personal aspects and factors of each woman’s life context are also directly involved in the preparation and delivery of the child’s food, insofar as she interprets and adjusts the official recommendations according to her reality^(^ 20 ^)^, which may have influenced the gradual decline in breastfeeding over the months among the participants.

In the analysis of associations between the sociodemographic variables and EBF duration at 30, 60, and 180 days after delivery, the “religion” variable had an influence on maintaining this practice at 30 and 60 days after delivery. Considering that individual and population health determinants bring together customs, beliefs, and values, and that this includes religious culture, the choice of women to breastfeed may or may not be influenced by religion or by adhering to traditional or modern values of a given population^(^ 21 ^)^. Similarly to the present study, an American study involving 4,898 mother-child binomials belonging to the disadvantaged economic class showed that religious affiliation was strongly associated with the beginning of breastfeeding, but weakly associated with its maintenance^(^ 22 ^)^. Thus, the formation of support and incentive networks that include churches and religious spaces is an essential strategy to strengthen healthy attitudes and behaviors among the population, including breastfeeding^(^ 22 ^)^.

This study also found a significant association between exclusive breastfeeding at 60 days and assistance to care for the baby, corroborating a study that demonstrates the valuable role of people who support and help women in the postpartum period, even though they are laypeople, which favors breastfeeding^(^ 8 ^)^.

Finally, maternal breastfeeding self-efficacy did not show a statistically significant association with the duration of EBF in the periods of 30, 60 and 180 days after delivery. However, a number of studies have already shown that there is a positive relationship between high self-efficacy and the duration of EBF for a longer time^(^ 10 ^,^ 16 ^)^. The high level of self-efficacy in the postpartum period seen in the present study shows the importance of verbal persuasion for self-efficacy, as this result may have been a reflection of the BFHI actions that are undertaken by the professionals who work in the maternity ward where the study took place. It is worth remembering that the institutions accredited by the BFHI have a strong role in actions in favor of breastfeeding. This initiative favors breastfeeding, increasing the rate of initiation and maintenance of this practice, with a positive impact on breastfeeding results in the short, medium, and long term^(^ 23 ^)^. Thus, the answers of the participants may have been influenced by the pro-breastfeeding practices of motherhood, a relationship that needs further investigation.

The present study was limited by the impossibility of monitoring the women in person, which would even allow for the application of BSES-SF at each investigated moment and not only in the postpartum period. The telephone search was the way found to carry out the monitoring, being valid to achieve the objectives proposed for this research.

The results presented can help the health professional to identify women with a higher risk of early weaning, enabling a faster and more effective intervention. The importance of improving the quality of obstetric care is emphasized, such as the reduction of elective cesarean sections and the increase in normal birth rates, thus reducing complications in the postpartum period and favoring the construction of positive maternal self-efficacy, in addition to other benefits for maternal and neonatal health. The findings regarding religion and the assistance received by the participant also show the need for efforts by the health professionals to establish and strengthen support networks for women after hospital discharge, recognizing the role of the family and of religious entities. It is suggested that the health teams can strengthen the bond with the families and with the community support networks, in order to prepare them to receive the woman who breastfeeds.

## Conclusion

Self-efficacy is a variable that is easily accessible and subject to change, through guidelines and stimuli that strengthen the internal (personal motivation) and external (technical) aspects of breastfeeding. This study analyzed that there is a relationship between breastfeeding self-efficacy and the type of delivery, the absence of complications in the postpartum period, having a religion, and having help with baby care.

The associations of self-efficacy with vaginal delivery and with the absence of complications in the postpartum period reinforce the results that qualified delivery care is beneficial for both women and breastfeeding. In addition, the specificities verified among the participants, related to the religion and assistance with baby care variables as factors that influenced the duration of exclusive breastfeeding, demonstrate that these variables are important to support breastfeeding and deserve greater attention from the professionals who work to promote breastfeeding.

The possibility is also highlighted of further studies that investigate self-efficacy over the months, in order to study more deeply how this construct changes after hospital discharge, in the contextual reality of the woman and of her child.
